# Esophageal lichen planus: A prospective interdisciplinary, monocentric cohort study

**DOI:** 10.1111/ddg.15808

**Published:** 2025-08-02

**Authors:** Rebecca Diehl, Annette Schmitt‐Graeff, Dimitra Kiritsi, Wolfgang Kreisel, Arthur Schmidt, Annegrit Decker, Franziska Schauer

**Affiliations:** ^1^ Department of Dermatology Medical Center‐University of Freiburg Faculty of Medicine University of Freiburg Freiburg Germany; ^2^ Medical Center‐University of Freiburg Faculty of Medicine University of Freiburg Freiburg Germany; ^3^ First Department of Dermatology and Venereology Faculty of Medicine Aristotle University of Thessaloniki Thessaloniki Greece; ^4^ Department of Medicine II Gastroenterology Hepatology Endocrinology and Infectious Diseases Medical Center‐University of Freiburg Faculty of Medicine Freiburg Germany

**Keywords:** Dysphagia, bolus obstruction, denudation, trachealization, squamous cell carcinoma

## Abstract

**Background and objectives:**

Lichen planus (LP) is an inflammatory condition that affects skin, hair follicles, nails and mucous membranes with esophageal involvement being an underrecognized manifestation.

**Patients and Methods:**

In this prospective cohort study (2020–2023), we screened 562 patients for symptomatic and clinically relevant esophageal lichen planus (ELP), using dysphagia as the primary screening criterion. The study included patients from the Department of Dermatology with either newly diagnosed or existing LP who reported esophageal symptoms, as well as patients referred from the Department of Gastroenterology who underwent endoscopy for unexplained esophageal symptoms potentially associated with ELP. The diagnosis of ELP was based on endoscopic, histopathological, and immunofluorescence findings.

**Results:**

Through this approach, 77 patients with dysphagia and potential ELP were identified, of whom 21 were diagnosed with ELP. A more detailed evaluation of their symptoms revealed that ELP patients exhibited significantly higher rates of esophageal dysphagia, food bolus obstruction, and retrosternal pain compared to LP patients without esophageal involvement. Two ELP patients were diagnosed with esophageal squamous cell carcinoma. Multilocular LP manifestation emerged as a strong indicator of esophageal involvement.

**Conclusions:**

This study underscores the importance of recognizing ELP, recommending comprehensive endoscopic evaluation and dermatological assessments when esophageal symptoms are present.

## INTRODUCTION

Lichen planus (LP) is a widespread inflammatory condition affecting approximately 1.3% of the global population, with a higher incidence in women.^1^, ^2^, ^3^, ^4^ LP is characterized by interface dermatitis, driven by a type 1 immune response that leads to keratinocyte necroptosis.^5^ A key cytokine involved in this process is IFN‐y, produced by cytotoxic T cells, which acts on keratinocytes via JAK/STAT signaling pathway.^6^

LP presents in three primary forms, affecting the skin, skin, hair follicles, nails, and mucous membranes, and the manifestations may occur individually or in combination.^3^ Mucosal lichen planus can impact various body sites, including the mouth, throat, esophagus, genital area, and in rare cases, the eyes. Oral involvement is particularly common, occurring in approximately two‐thirds of cases with cutaneous involvement.^7^, ^8^ There is evidence suggesting a link between oral lichen planus (OLP) and hepatitis B (HBV) and C virus infections (HCV), which may acts as potential trigger.^9^ OLP is recognized as a pre‐malignant condition, with patients facing an increased risk of developing oral squamous cell carcinoma (SCC) due to chronic inflammation.^10^

Esophageal lichen planus (ELP) is one of several inflammatory esophageal conditions to consider in patients presenting with esophageal symptoms.^11^, ^12^ The differential diagnosis for such symptoms is broad and includes common conditions such as reflux esophagitis (GERD), as well as malignancies, infectious causes, immunological disorders such as eosinophilic esophagitis (EoE) or autoimmune bullous diseases.^13^, ^14^, ^15^, ^16^, ^17^ While cases of ELP without esophageal symptoms have been reported in the literature, most patients with clinically significant ELP who require treatment exhibit at least one extra‐esophageal symptom, such as nail involvement.^18^

ELP was long considered a rare condition, but it may often have been underdiagnosed due to a lack of specific diagnostic criteria within the broad spectrum of differential diagnoses. In recent years, diagnostic criteria have been proposed and applied to previous clinical cohorts.^19^, ^20^ While some recent studies suggest that esophageal involvement may occur in up to half of patients with cutaneous LP or OLP, the actual frequency of ELP may also be lower than reported due to small sample sizes and potential selection bias.^21^, ^22^, ^23^ In this study, we investigated this gastroenterological manifestation of LP in a prospective and multidisciplinary manner to further determine its clinical and diagnostic features.

## PATIENTS AND METHODS

From January 2020 to December 2023, we conducted a prospective cohort study in the Departments of Dermatology and Gastroenterology at the Medical Center, University of Freiburg. Patients were included via two pathways: through dermatology, with confirmed LP of the skin, hair follicules or nails and dysphagia (oropharyngeal or esophageal), or through gastroenterology, when referred for endoscopy to evaluate previously undefined esophageal disease likely consistent with ELP. After obtaining consent, the attending physician completed collection of data on personal history and clinical manifestation. The localization of LP was assessed in the following areas: oral, genital, anal, eye, skin, esophagus and nails. Endoscopy was conducted and analyzed using defined macroscopic criteria (Table 1).^20^ The included patients completed questionnaires that provided detailed information about their esophageal symptoms, such as any dysphagia, heartburn, bolus obstruction, regurgitation and unwanted weight loss.

**TABLE 1:** Diagnostic criteria and severity grading system for ELP, as previously described.^20^

Macroscopic signs in endoscopy (D regarded as specific sign and H, T, S as possible signs)		
*Denudation of the mucosa (D)*: D1: iatrogenic D2: spontaneous, localized (< 1 cm^2^) D3: spontaneous, localized (> 1 cm^2^)	*Hyperkeratosis (H*) *Trachealization (T)*	*Stenosis (S)*: S1: endoscope can pass S2: endoscope cannot pass or diameter < 1 cm
**Microscopic histopathological criteria (HP): One point each: HP0 (negative) to HP3 (strong positive**)		
Epithelial detachment Lymphocytic infiltrate Intraepithelial apoptosis (Civatte bodies) Dyskeratosis		
**Direct immunofluorescence**		
*Fibrinogen (F)* F0: none F1: weak positive F2: positive		
**Severity grading system for ELP**		
*Severe ELP*: ≥ D2 and HP ≥ 1 and/or F ≥ 1 *Mild ELP: 1) D1 and HP ≥ 1 and/or F ≥ 1 2) S, H, T or no endoscopic sign and HP1 ≥ + F ≥ 1* *No ELP: none of the above‐mentioned criteria are fulfilled*		

*Abbr*.: D, denudation; DIF, direct immunofluorescence; F, fibrinogen deposit in DIF; H, hyperkeratosis; HP, histopathology; S, stenosis; T, trachealization

For histopathological analysis, tissue biopsies were taken from both the lower and upper thirds of the esophagus. To minimize interference with potential histopathological features of a co‐existing GERD, biopsies were not taken within approximately 5 cm of the gastroesophageal junction or from areas showing reflux‐related lesions. Additionally, a third biopsy from the mid‐esophagus was taken for direct immunofluorescence microscopy (DIF). Tissue samples for standard histopathology were preserved in formalin, embedded in paraffin and stained with hematoxylin and eosin, Giemsa and periodic acid‐Schiff. DIF was performed on thin sections of fresh frozen tissue using antibodies against IgG, IgA, IgM, C3c and fibrinogen, following an established protocol.^20^ For ELP diagnosis, we used the previously proposed diagnostic criteria (Table 1). Serological screening for HBV and HCV infection was also conducted.

REDCap, a secure, web‐based platform provided by the University of Freiburg, facilitated standardized and systematic data collection for the patient cohort research.^24^, ^25^ Subsequently, the data was anonymized and analyzed using the R Studio software no. 2024.04.1+748. The t‐test was used to compare numeric and binary variables. The Fisher's exact test was applied to compare binary and grouped variables. P values < 0.5 were considered statistically significant. The study was approved by the Ethic Committee in Freiburg with Number 20‐1227‐1. The study was registered in the German Clinical Trials Register (Deutsches Register Klinischer Studien, DRKS) with the registration number: DRKS00023700. The manuscript was structured according to the STROBE checklist.^26^

## RESULTS

### Demographics and group characterisation

**FIGURE 1:**
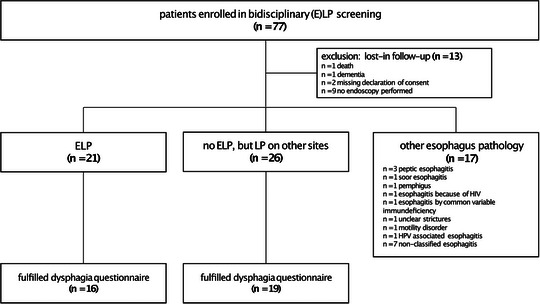
Patient recruitment: A total of 77 patients were included in the study. Individuals were referred from gastroenterology with suspected esophageal lichen planus (ELP) requiring dermatological co‐evaluation, as well as patients from dermatology with confirmed lichen planus (LP) of any side who reported dysphagia, warranting endoscopic evaluation for ELP. Thirteen patients were excluded due to loss of follow‐up, and 17 patients were found to have other esophageal pathologies. Among the remaining 47 patients, 21 were diagnosed with ELP, while 26 had LP in other locations but no esophageal involvement.

**FIGURE 2:**
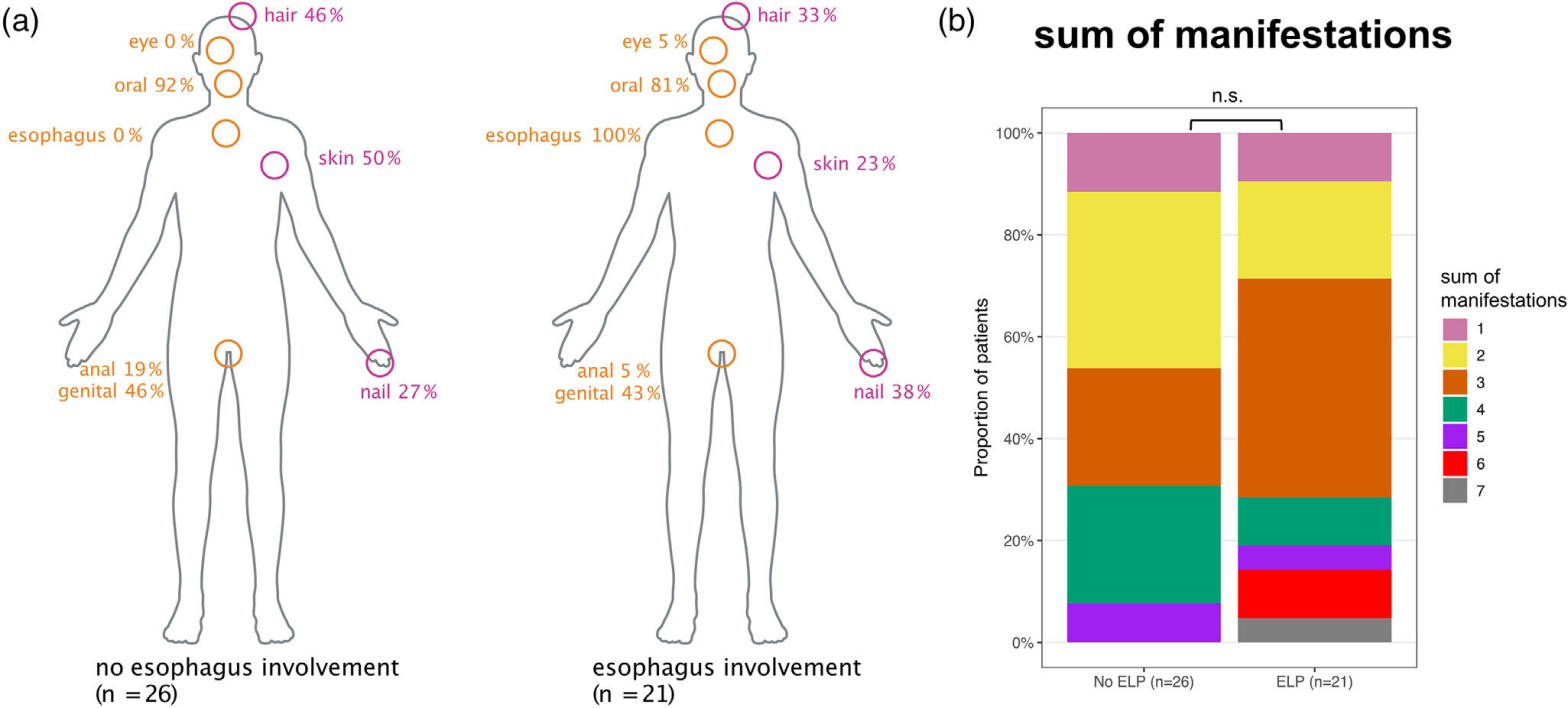
(a) Distribution of LP side manifestations in patients without ELP (n = 26) and with ELP (n = 21). While skin and anal involvement were less frequent in the ELP group compared to the non‐ELP group, these differences did not reach statistical significance. (b) Comparison of the total number of LP side manifestations between non‐ELP (n = 26) and ELP (n = 21) groups. While no significant difference was observed between groups (p > 0.05), it is noteworthy that all three patients with the highest number of manifestations (6 or 7) were in the ELP group.

We evaluated 562 patients with LP for symptomatic and clinically relevant ELP. Through this approach, we included and collected data from 77 patients (n = 50 from dermatology, n = 27 from gastroenterology) with suspected or potential ELP (Figure 1). Thirteen patients were excluded because of dementia, death, or withdrawal of consent for endoscopy. In 17 out of the remaining 64 patients, no LP manifestations were found by endoscopic, histopathological or dermatologic examinations and were not considered for futher analysis. Twenty‐one patients were diagnosed with ELP (ELP group), while 26 patients had dysphagia without any confirmed esophageal pathology, but LP in other areas (non‐ELP group) (Figure 2). The final analysis therefore included 47 patients with a confirmed LP diagnosis at any site based on the dermatologic and endoscopic assessment.

All ELP cases met the full diagnostic criteria previously published^20^ (online supplementary Table S1, Nos. 1–21), with two patients meeting the criteria for severe ELP (online supplementary Table S1, Nos. 7–8), and two patients presenting with isolated ELP (online supplementary Table S1, Nos. 17–18). In a total of 562 LP patients treated at our medical center in the study period, we identified symptomatic ELP in 3.7% of cases.

The median age in the ELP group was 72 years, compared to 57 years in the non‐ELP group. Women were more frequently affected in both groups, comprising 71% of the ELP group and 88% of the non‐ELP group. The median body mass index (BMI) was lower in the ELP group (24 kg/m^2^) compared to the non‐ELP group (26 kg/m^2^). Smoking rates were similar in both groups, with 33% in the ELP group and 38% in the non‐ELP group (Table 2).

**TABLE 2:** Patient characteristics.

	ELP (n = 21)	No ELP (n = 26)
Mean age [years]	72 [q1: 65; q3: 80]	57 [q1: 46; q3: 67]
Women [%]	71% (15/21)	88% (23/26)
Mean BMI [kg/m^2^]	24 [q1: 22; q3: 26] (9 NA)	26 [q1: 22; q3: 29] (14 NA)
Smoking	33% (5/15, 6 NA)	38% (6/16, 10 NA)
Hepatitis C virus infection	5% (1/20)	4% (1/26)
Hepatitis B virus infection	5% (1/20)	8% (2/26)
Developing esophageal SCC	10% (2/20)	0% (0/26)

*Abbr*.: BMI, body mass index; ELP, esophageal lichen planus; NA, not available; q1, first quartile; q3, third quartile; SCC, squamous cell carcinoma

A history of HCV infection was present in 5% of the ELP group and 4% of the non‐ELP group. In the ELP group, this referred to patients who had previously been successfully treated for HCV‐infection, whereas in the non‐ELP group, one patient was newly diagnosed with HCV infection based on our screening. Regarding HBV infection, there was one case of chronic HBV infection in each group, along with an additional case of resolved HBV infection in the non‐ELP group. Notably, two of the 21 patients in the ELP group were diagnosed with esophageal SCC at UICC stage I, whereas no cases of SCC were detected in the non‐ELP group (Table 2).

### Endoscopic and histological findings

During endoscopy, mucosal denudation, a specific macroscopic finding, was observed in 62% of patients, with most cases classified as D1 (52%) and only two patients (10%) presenting with D2, which is considered severe ELP according to the grading system.^20^ Non‐specific findings were common, with 81% of the patients showing at least one finding, including 42% displaying hyperkeratosis and 71% showing trachealization. Endoscopically passable stenosis (S1) was observed in 29% of the patients, while 33% had S2 stenosis; and 29% required endoscopic dilation. Esophageal candidiasis was diagnosed in 19% of patients, likely as a side effect of topical steroid treatment. In terms of microscopic findings, 90% of patients exhibited characteristic histological features of ELP. 92% of those who provided adequate material for analysis had positive DIF results (fibrinogen only, Table 3).

**TABLE 3:** Endoscopic, histological, and immunofluorescence findings in the ELP group.

Endoscopic criteria ELP (n = 21)			
Denudation		13/21 (62%)	
	D1		11 (52%)
	D2		2 (10%)
	D3		0 (0%)
Non‐specific findings		17/21 (81%)	
	Hyperkeratosis		9 (42%)
	Trachealization		15 (71%)
	Stenosis		13/21 (62%)
	S1		6 (29%)
	S2		7 (33%)
**Histological findings ELP (n = 21)**			
*Characteristic ELP histopathology ≥ HP1*		*19/21 (90%)*	
	Civatte bodies		7 (33%)
	Dyskeratosis		12 (57%)
	Detachment of epithelium		7 (33%)
	Lymphocytic infiltrate		16 (76%)
**Direct immunofluorescence (n = 14, 7 NA)**			
Positive fibrinogen deposit in DIF ≥ F1		13/14 (92%)	
	F1 (weak)		5 (35%)
	F2 (strong)		8 (57%)

*Abbr*.: DIF, direct immunofluorescence; ELP, esophageal lichen planus; HP, histopathology; NA, not available; SCC, squamous carcinoma; S, stenosis

The most common additional clinical LP manifestation site in both groups was oral LP (81% ELP group, 92% non‐ELP group) (Figure 2a). Skin and anal involvement was less frequent in ELP than in non‐ELP cases, though the differences were not statistically significant. Other manifestations (nail, hair, genital) occurred with similar frequency in both groups. There was no statistically significant difference in the total number of manifestations between patients with ELP and those without. However, it is notable that three patients with the highest number of manifestations – six or seven – were all diagnosed with ELP, though the sample size is too small to draw robust conclusions (Figure 2b). On the other hand, two ELP patients had no LP manifestation at any other site than the esophagus.

### Dysphagia questionnaire findings

**FIGURE 3:**
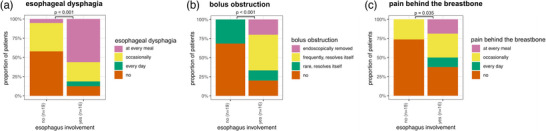
Clinical questions significantly correlating to ELP:(a) esophageal dysphagia; (b) bolus obstruction; (c) retrosternal pain.

We further investigated the esophageal symptoms reported by patients with and without confirmed diagnosis of ELP. The questionnaires were completed by 19 patients in the non‐ELP group and by 16 patients in the ELP group. Significant differences (p < 0.05) were found between the two groups in three key areas: *(1)* Esophageal dysphagia: 56% of ELP patients reported difficulties swallowing with every meal, compared to only 5% of those without ELP (Figure 3a). *(2)* Bolus obstruction: In the ELP group, 44% regularly experienced food impaction, and 19% had required endoscopic removal of a food bolus at least once in the past. In contrast, none of the patients without ELP reported regular food impaction or required endoscopic bolus removal (Figure 3b). *(3)* Retrosternal pain: 19% of ELP patients experienced retrosternal pain with every meal, a symptom not reported by any patients in the non‐ELP group (Figure 3c).

## DISCUSSION

We here present data from a prospective cohort screening for clinically relevant ELP using a bidisciplinary approach. For diagnosis of ELP, we applied previously published criteria, comprising endoscopic, histologic, and DIF assessment.^23^ Using this approach, we identified 21 LP patients with ELP and 26 patients without esophageal manifestation (both with dysphagia) and could compare clinical details of the two groups. As an amendment to our previous data, clinical severity in ELP did not necessarily correlate strictly with the extent of mucosal denudation, a typical criterion used by gastroenterologists.^20^ Instead, severity appeared to include additional factors such as the degree of histological inflammation, clinical symptoms, and complications such as stenosis. Therefore, we recommend avoiding the previous subdivision into “mild” and “severe” forms of ELP based on mucosal denudation, as it complicates rather than facilitates therapeutic decision‐making.

Esophageal dysphagia encompasses a broad spectrum of diseases, including malignancy, peptic strictures due to reflux esophagitis, infections, motility disorders, and inflammatory diseases.^16^, ^18^, ^27^ Diagnosing ELP is challenging due to its symptomatic similarity to these conditions. Eosinophilic esophagitis can be clearly distinguished from ELP histologically, with EoE showing more than 15 eosinophils per high‐power field.^28^ GERD can be differentiated from ELP via endoscopy or, in unclear cases, through a combination of endoscopy and histology.^23^ Some experts consider ELP and lichenoid esophagitis to be variations of lymphocytic esophagitis, though ELP histologically exhibits apoptotic keratinocytes, known as Civatte bodies, which are typically absent in lichenoid esophagitis.^16^, ^27^, ^29^ Although lichenoid esophagitis may clinically resemble ELP, the latter is more frequently associated with the development of esophageal stenosis.^27^ We found that it is crucial for ELP diagnostics to be conducted by physicians experienced with the condition. Although histological evaluation did not always contribute to the diagnosis, review by an experienced pathologist helped improve diagnostic accuracy. In order to be able to assess histological findings consistent with ELP, pathologists need to have samples containing esophageal stroma and not only detached epithelium. Ensuring sufficient biopsy depth is therefore crucial in clinical practice. In our cohort, this was not an issue, likely due to the expertise of the investigators, highlighting the importance of clinician awareness and training. In our experience, diagnostic accuracy improves when all diagnostic components (macroscopic, histologic and DIF assessment) are included. However, external diagnostic workup was sometimes incomplete as DIF microscopy was never performed. Since the diagnostic criteria may not be sufficiently known among gastroenterologists and pathologists, cases may be underreported, potentially leading to false‐negative diagnoses. This highlights the critical need for knowledge transfer about ELP within the medical community. The diagnosis can be accurately established through interdisciplinary consultation involving gastroenterologists, dermatologists, and pathological experts.

In our cohort, patients diagnosed with ELP were generally older than those without ELP, and women were more frequently affected, accounting for 71% of the cases, which aligns with previous reports in the literature.^30^ Patients with LP but without ELP most frequently had oral involvement, indicating that their reported dysphagia was oropharyngeal rather than esophageal in nature. There was no significant difference in the prevalence of manifestation sites between the groups, although there was a tendency for skin and anal involvement to be less common in ELP patients. The number of LP manifestations did not differ between the two groups, However, patients with the highest number of manifestations (6 or above) were all diagnosed with ELP, suggesting that individuals with multilocular disease activity may be at a higher risk of developing ELP. This highlights the importance of an interdisciplinary approach in diagnosing and managing (E)LP, recognizing that the likelihood of esophageal involvement is higher in patients with LP at other sites. It also underscores the need for dermatological evaluations to prevent false negative interpretations of endoscopic and histological findings.^20^ Consistent with previous studies, we found that ELP can occasionally also present as the sole manifestation of the disease, as observed in two patients in our cohort.^20^

Based on these considerations and our experience in applying the published criteria, we propose a revised and simplified approach for diagnosis of ELP. The diagnosis could be established in the following scenarios: *(1)* Endoscopic denudation (D) of any grade, accompanied by either a positive histopathological finding, or positive DIF for fibrinogen depositions. *(2)* At least one endoscopic sign (S, H, T), along with either a positive histopathological result or positive DIF result, or along with dermatological confirmation of LP manifestation in other areas and histological exclusion of other common esophageal differential diagnoses (online supplementary Table S2).

Two patients in the ELP group (10%) were diagnosed with esophageal SCC, highlighting the risk of SCC development in this group, which is reported to be 5–6% in the literature.^18^, ^31^ This underscores the importance of endoscopic follow‐up in patients with known ELP to detect potential SCC transformation at an early stage.^18^, ^31^ One of these patients had a history of chronic HCV infection, which had been cured with antiviral treatment several years prior to SCC diagnosis. The infection possibly served as a trigger for ELP, while persistent chronic inflammation may have contributed to the development of SCC. However, there was no significant difference in the prevalence of HBV and HCV infections between the ELP and non‐ELP groups.

Of the 64 patients that underwent bidisciplinary examinations in our study for suspected ELP, 17 patients were ultimately not diagnosed with any LP manifestation. This was due to the study design, allowing screening of gastroenterological patients with high probability of ELP characteristics or lichenoid esophagitis pattern. Of the 47 remaining patients, all of whom had confirmed LP at one or more sites and who presented with esophageal symptoms, 44.6% (21/47) were diagnosed with ELP. When considering the total of 562 patients treated for any diagnosed LP manifestation at our center in the 4‐year study period, we diagnosed ELP in 3,7% (21/562) of the cases using the symptom‐based screening approach. This risk may be underestimated due to the exclusion of 13 symptomatic patients who were lost to follow‐up and did not undergo endoscopy. Whether this represents a true prevalence remains speculative due to the pre‐selected patient population. Given the study design, our conclusions must be interpreted with caution. A prospective, multicenter study with systematic screening of asymptomatic patients is needed to determine the actual prevalence and further refine the characterization of the disease. These results underline that although clinically manifest ELP appears to be a rare condition in the general population, esophageal involvement should be suspected in up to 45% of patients with known LP who report dysphagia. Endoscopic screening of this pre‐selected group should therefore be recommended in clinical practice.

We demonstrated that patients with ELP showed three distinct symptoms compared to the non‐ELP group: *(1)* frequent esophageal dysphagia with problems swallowing at every meal; *(2)* frequent food bolus obstruction, sometimes requiring endoscopic removal; *(3)* frequent retrosternal pain. These findings also suggest that a targeted questionnaire focusing on these specific symptoms could serve as a screening tool for dermatologists to identify any LP patients requiring endoscopic screening for ELP.

However, due to preselection and the design of the current study, we may have overlooked patients with asymptomatic ELP.^21^, ^22^ Although relatively rare, asymptomatic cases have been reported.^19^, ^20^ For instance, Fox et al. reported that 17% of 72 patients with ELP were either asymptomatic or had only minimal symptoms.^18^ Future studies should consider these factors and aim to include asymptomatic cases. While diagnosing ELP in asymptomatic patients may be considered primarily an academic exercise, treatment decisions should be guided by the presence of clinical symptoms, as well as endoscopic and histological findings.^23^ Nonetheless, identifying such cases may be crucial due to the potentially increased risk of developing esophageal SCC. However, given economic and risk considerations, it appears impractical to perform routine endoscopy for SCC screening in all patients with LP at any site, particularly in the absence of esophageal symptoms. For the same reasons, esophageal cancer screening is not established for the general population in low‐prevalence countries, such as Germany.^32^ We propose that a targeted questionnaire addressing key esophageal symptoms could serve as an effective pre‐screening tool for LP patients. This would help determine whether endoscopy is necessary, striking a favorable balance between benefit and risk.

We recommend treating ELP in cases of dysphagia, endoscopic or histological signs of inflammation and/or stenosis. The primary goal is to alleviate symptoms, to address stenosis, and prevent long‐term complications. A unique characteristic of ELP is that stenosis can regress with anti‐inflammatory therapy, often eliminating the need for dilation.^20^ Additionally, such therapy might prevent long‐term consequences of chronic active inflammation, such as scarring stenosis or even carcinoma development. At our center, topical budesonide has proven to be the most effective treatment, administered either as a viscous solution or as orodispersible tablets originally developed for conditions such as EoE (online supplementary Table S3).^20^, ^33^ Moreover, T‐cell‐depletion‐medication, biologicals, and small molecules are currently being used in ELP treatment. JAK‐Inhibitors represent the most promising therapeutic alternative.^34^, ^35^ It is important to note that all of these treatment options are currently used off‐label.^36^

## FUNDING

R.D. is funded by the Deutsche Forschungsgemeinschaft (DFG, German Research Foundation) – CRC1160/2 – B03(N), Medical Center – University of Freiburg, and Faculty of Medicine, University of Freiburg, and supported by the Berta‐Ottenstein‐Programme for Clinician Scientists, Faculty of Medicine, University of Freiburg. F.S. was supported by the Berta‐Ottenstein‐Programme Advanced Clinician Scientist of the Faculty of Medicine, University of Freiburg.

## CONFLICT OF INTEREST STATEMENT

None.

## Supporting information



Supplementary information
