# Supplementary material for: Esophageal lichen planus: A prospective interdisciplinary, monocentric cohort study
Source: J Dtsch Dermatol Ges. 2025 Aug 2;23(11):1394–401. doi: 10.1111/ddg.15808 (PMC12619036; doi:10.1111/ddg.15808)
Supplement: Supplementary file 1 — Supplementary information [file DDG-23-1394-s001.docx]

[[online supplement]]

Online supplementary TABLE S1: Endoscopic, histological, immunofluorescence, and clinical characteristics of ELP patients.

| **No.** | **Age** | **Sex** | **H** | **T** | **D1** | **D2** | **D3** | **S1** | **S2** | **Dilation** | **Soor** | **HP** | **Civatte bodies** | **Dyskeratosis** | **Detachment of the epithelium** | **Lymphocytic (T-cell) infiltrate** | **F0** | **F1 (weak)** | **F2 (strong)** | **Endoscopic + histology + DIF** | **Oral** | **Genital** | **Anal** | **Skin** | **Hair** | **Nail** | **Eye** |
| --- | --- | --- | --- | --- | --- | --- | --- | --- | --- | --- | --- | --- | --- | --- | --- | --- | --- | --- | --- | --- | --- | --- | --- | --- | --- | --- | --- |
| 1 | 85 | m | No | Yes | Yes | No | No | No | Yes | Yes | No | HP3 | No | Yes | Yes | Yes | No | No | Yes | T, D1, S2, Dilation, HP3, F2 | Yes | No | No | No | No | No | No |
| 2 | 63 | f | No | Yes | No | No | No | No | Yes | No | Yes | HP3 | No | Yes | Yes | Yes | No | Yes | No | T, S2, HP3, F1 | Yes | Yes | No | Yes | Yes | Yes | No |
| 3 | 68 | f | Yes | Yes | Yes | No | No | Yes | No | No | Yes | HP3 | Yes | No | Yes | Yes | No | No | Yes | H, T, D1, S1, HP3, F1 | Yes | No | No | No | Yes | No | No |
| 4 | 75 | m | No | Yes | No | No | No | No | Yes | No | No | HP2 | Yes | No | No | Yes | No | No | Yes | T, S2, HP3, F2 | Yes | No | No | No | Yes | No | No |
| 5 | 80 | m | No | Yes | Yes | No | No | No | Yes | Yes | No | HP3 | No | Yes | Yes | Yes | Yes | No | No | T, D1, S1, Dilation, HP3, F0 | Yes | No | No | No | Yes | Yes | No |
| 6 | 70 | f | No | No | No | No | No | Yes | No | No | Yes | HP3 | No | Yes | Yes | Yes | No | No | Yes | S1, HP3, F2 | Yes | No | No | No | No | Yes | No |
| 7 | 66 | f | Yes | Yes | No | Yes | No | No | Yes | Yes | No | HP2 | Yes | Yes | No | No | No | Yes | No | H, T, D2, S2, Dilation, HP2, F1 | Yes | Yes | No | Yes | Yes | No | No |
| 8 | 79 | f | Yes | Yes | No | Yes | No | Yes | No | Yes | No | HP2 | No | Yes | No | Yes | NA | NA | NA | H, T, D2, S1, Dilation, HP2, FNA | Yes | Yes | Yes | Yes | No | Yes | No |
| 9 | 83 | f | No | No | Yes | No | No | No | No | No | No | HP1 | No | No | No | Yes | NA | NA | NA | D1, HP1, FNA | Yes | No | No | No | No | Yes | No |
| 10 | 74 | f | Yes | Yes | No | No | No | No | No | No | No | HP1 | No | No | No | Yes | No | No | Yes | H, T, HP1, F2 | Yes | Yes | No | No | No | No | No |
| 11 | 56 | f | Yes | Yes | Yes | No | No | No | No | No | No | HP0 | No | No | No | No | No | No | Yes | H, T, D1, HP0, F2 | Yes | No | No | Yes | No | No | No |
| 12 | 63 | f | No | No | Yes | No | No | No | No | No | No | HP0 | No | No | No | No | No | Yes | No | D1, HP0, F1 | Yes, gum retraction | Yes | No | No | No | No | No |
| 13 | 87 | m | No | No | No | No | No | No | No | No | No | HP1 | No | No | No | Yes | No | No | Yes | HP1, F2 | Yes | No | No | No | No | No | No |
| 14 | 65 | f | No | No | Yes | No | No | No | No | No | No | HP2 | Yes | No | No | Yes | No | No | Yes | D1, HP2, F2 | Yes | Yes | No | No | No | No | No |
| 15 | 87 | f | Yes | Yes | No | No | No | Yes | No | Yes | No | HP2 | No | Yes | No | Yes | NA | NA | NA | H, T, S1, HP2. FNA | Yes | No | No | No | No | Yes | No |
| 16 | 75 | f | Yes | Yes | Yes | No | No | No | No | No | No | HP3 | Yes | Yes | Yes | No | NA | NA | NA | H, T, D1, HP3, FNA | Yes | Yes | No | No | No | No | No |
| 17 | 75 | f | No | Yes | Yes | No | No | No | Yes | No | No | HP2 | Yes | No | No | Yes | No | Yes | No | T, D1, S2, HP2, F1 | No | No | No | No | No | No | No |
| 18 | 62 | f | Yes | Yes | Yes | No | No | Yes | No | No | Yes | HP3 | Yes | Yes | Yes | Yes | No | Yes | No | H, T, D1, S1, HP3, F1 | No | No | No | No | No | No | No |
| 19 | 79 | m | Yes | Yes | No | No | No | No | No | No | No | HP2 | No | Yes | No | Yes | NA | NA | NA | H, T, HP2, FNA | Yes, gum retraction | No | No | No | No | Yes | No |
| 20 | 45 | f | No | Yes | No | No | No | No | Yes | Yes | No | HP1 | No | Yes | No | No | NA | NA | NA | T, S2, Dilation, HP1, FNA | Yes | Yes | No | Yes | No | Yes | Yes |
| 21 | 81 | f | No | No | Yes | No | No | Yes | No | No | No | HP1 | No | No | No | Yes | NA | NA | NA | D1, S1, HP1 | Yes | Yes | No | No | Yes | No | No |

*Abbr.:* D, denudation; DIF, direct immunofluorescence; F, fibrinogen deposit in DIF; H, hyperkeratosis; HP, histopathology; NA, not available; S, stenosis; SCC, squamous cell carcinoma; T, trachealization

Online supplementary TABLE S2: Comparison of previous and adapted diagnostic criteria. The adapted first criterion consolidates the former two criteria, eliminating the previous classification of "mild" and "severe" forms based on mucosal denudation extent and hereby simplifying diagnosis. The previously existing criterion 3 has been expanded to emphasize the importance of comprehensive dermatological examination, recognizing that other lichen planus manifestations can provide valuable diagnostic insights, especially in cases with subtle or nonspecific endoscopic and histological findings

| Previous diagnostic criteria | Adapted diagnostic criteria |
| --- | --- |
| 1. ≥ D2 and HP ≥ 1 and/or F ≥ 1, considered as a severe case of ELP 2. D1 and HP ≥ 1 and/or F ≥ 1 3. S, H, T or any case with no/unclear endoscopic signs, but HP ≥ 1 and F ≥ 1 | 1. ≥ D1 and HP ≥ 1 and/or F ≥ 1 2. S, H, T and one of the following:  - HP ≥ 1 or - F ≥ 1 or - dermatological confirmation of LP manifestation in other areas and histological exclusion of other common esophageal differential diagnoses |

*Abbr.:* D, denudation; DIF, direct immunofluorescence; F, fibrinogen deposit in DIF; H, hyperkeratosis; HP, histopathology; LP, lichen planus; S, stenosis; T, trachealization

Online supplementary TABLE S3: ELP patients, therapies, and treatment outcomes.

| **No.** | **Age** | **Sex** | **Endoscopic + histology + DIF** | **Therapy** | **Outcome** |
| --- | --- | --- | --- | --- | --- |
| 1 | 85 | M | T, D1, S2, Dilation, HP3, F2 | Topical budesonide | Reduction of inflammation, improvement of S |
| 2 | 63 | F | T, S2, HP3, F1 | Topical budesonide, orodispersable tablets | Reduction of inflammation, improvement of S |
| 3 | 68 | F | H, T, D1, S1, HP3, F1 | Topical budesonide | complete remission of S and T |
| 4 | 75 | M | T, S2, HP3, F2 | Topical budesonide | Minimal improvement of S and T |
| 5 | 80 | M | T, D1, S1, Dilation, HP3, F0 | Topical budesonide | Reduction of inflammation, recurrent thrush esophagitis, improvement of S |
| 6 | 70 | F | S1, HP3, F2 | Topical budesonide | No repeat endoscopy in our center |
| 7 | 66 | F | H, T, D2, S2, Dilation, HP2, F1 | Topical budesonide, mycophenolate-mofetil, cyclophosphamide, tofacitinib | No long-term improvement with budesonide, mycophenolate-mofetil and cyclophosphamide; with tofacitinib reduction of inflammation, H, T, D and improvement of S |
| 8 | 79 | F | H, T, D2, S1, Dilation, HP2, FNA | Topical budesonide | Reduction of inflammation, improvement of S, D, T |
| 9 | 83 | F | D1, HP1, FNA | Topical budesonide | No repeat endoscopy in our center |
| 10 | 74 | F | H, T, HP1, F2 | Topical budesonide | Minimal improvement of S and T |
| 11 | 56 | F | H, T, D1, HP0, F2 | Methotrexat, mycophenolate-mofetil | Reduction of inflammation |
| 12 | 63 | F | D1, HP0, F1 | Systemic steroid, methotrexate | Reduction of inflammation |
| 13 | 87 | M | HP1, F2 | No treatment | No treatment |
| 14 | 65 | F | D1, HP2, F2 | Topical budesonide, guselkumab, mycophenolate-mofetil, baricitinib | No effect of topical budesonide and mycophenolate-mofetil, guselkumab: secondary loss of effect, baricitinib: improvement of inflammation and D |
| 15 | 87 | F | H, T, S1, HP2. FNA | Topical budesonide | Reduction of inflammation, improvement of S, T |
| 16 | 75 | F | H, T, D1, HP3, FNA | Topical budesonide, orodispersable tablets | No repeat endoscopy in our center |
| 17 | 75 | F | T, D1, S2, HP2, F1 | Topical budesonide | Reduction of inflammation, recurrent thrush esophagitis, improvement of S, T, D |
| 18 | 62 | F | H, T, D1, S1, HP3, F1 | Topical budesonide, orodispersable tablets | complete remission of H, S, T |
| 19 | 79 | M | H, T, HP2, FNA | NA | NA |
| 20 | 45 | F | T, S2, Dilation, HP1, FNA | Mycophenolate-mofetil | No repeat endoscopy in our center |
| 21 | 81 | F | D1, S1, HP1 | Topical budesonide | No repeat endoscopy in our center |

*Abbr.:* D, denudation; DIF, direct immunofluorescence; F, fibrinogen deposit in DIF; H, hyperkeratosis; HP, histopathology; LP, lichen planus; NA, not available; S, stenosis; T, trachealization
